# 65. Blau syndrome treated with sequential biologics

**DOI:** 10.1093/rap/rky034.028

**Published:** 2018-09-20

**Authors:** Cee Yi Yong, Chetan Mukhtyar, Kate Armon

**Affiliations:** 1 Rheumatology, Norfolk and Norwich University Hospital, Norwich, UNITED KINGDOM; 2 Paediatric Rheumatology, Norfolk and Norwich University Hospital, Norwich, UNITED KINGDOM

**Introduction:** Blau syndrome is a rare autosomal dominant autoinflammatory disease characterised by early-onset granulomatous arthritis, uveitis and rash. It is associated with NOD2/CARD 15 gene mutations. In some literature, Blau syndrome has been described as both the familial/inherited and sporadic form, while others refer Blau syndrome to the familial form and early onset sarcoidosis to the sporadic form. There is also a suggestion for the term juvenile systemic granulomatosis to include both Blau syndrome and early onset sarcoidosis. We present here a case of sporadic Blau syndrome confirmed by molecular genetic analysis. There is limited literature evidence to inform treatment. We report the control of this disease initially with anakinra for 12 years, and later with infliximab, resulting in successful controlled for the past 19 months and our patient entering full-time employment.

**Case description:** Our patient first presented at the age of one in 1991 to the dermatologist with an unusual skin eruption that started when he was two months old. His parents described a circular patch on his buttock that spread outwards with a firm centre that felt like a pea. He would have similar lesions in other parts of his body, which resolved spontaneously. The dermatologist described his skin rashes as unusual; consisting of reticulate erythema with superficial scaling and a more widespread doughy quality. Otherwise his physical examination was normal even though his parents were concerned that he could not grip easily and his fingers were slightly tender. His height was below the third centile and his weight was above the tenth centile. He had sustained a fractured skull secondary to trauma at five months old and had croup at one year old. There was no significant family history of skin or joints problems. Initial investigations showed normal full blood count, erythrocyte sedimentation rate (ESR) and serum calcium. Chest x-ray was normal. X-ray of the hands showed widening of tubular bones of the hand with a coarse trabecular pattern. A skin biopsy showed non-necrotising granulomatous inflammatory infiltrates associated with the hair follicles. Stains for fungi and mycobacteria were negative. Polarisation microscopy revealed tiny birefringent foreign material, some of which appeared to be epitheloid cells. This was reported as possibility of sarcoid however the presence of foreign material was unusual. At the age of 18 months, he was admitted to paediatric ward for further investigations as he developed swelling of his hands and feet. His rashes were persistent but he had no other symptoms. Clinically, his wrists, metacarpophalangeal, proximal interphalangeal joints and metatarsophalangeal joints appeared swollen. He was reviewed by ophthalmologist and found to have posterior synechiae on the left along with keratic precipitates and mild uveitis. His serum angiotensin-converting enzyme was raised at the level of 74 U/L (normal range 12-68 U/L). Rheumatoid factor, Anti-nuclear antibodies and Mantoux test were negative. He was diagnosed with juvenile sarcoidosis (Blau syndrome triad of arthritis, uveitis and rash) and commenced on 15mg prednisolone. His skin rashes and joints improved. However, his hands became swollen when the dose was reduced to 5mg on alternate days. He remained on 7.5mg (0.75mg/kg) prednisolone on alternate days with episodes of worsening joints swelling intermittently, which required increased steroid dose and addition of diclofenac. At the age of three, due to ongoing rashes and progressing destructive arthritis; subcutaneous methotrexate was added to prednisolone 10mg on alternate days. Methotrexate was gradually increased to maximum tolerable dose with the limitation of raised transaminase. Although there was an improvement in his joints and rashes, he was unable to wean off prednisolone below 7mg on alternate day. He had considerable joint stiffness on steroid off day. He also required intra-articular steroid injections. At the age of eight, he was referred to an endocrinologist for growth retardation, cushingoid features, hypertension and weight gain. At the age of nine, he developed left sided pan uveitis that required a short-term increase dose of prednisolone to 40mg. He had developed boutonniere deformity of both fourth fingers without active synovitis. The x-ray of the hands showed proximal metacarpal swelling progressing to lytic lesions at distal ends of proximal phalanges of all fingers. A DEXA scan showed a Z-score of -2.1. He was started on oral calcium and vitamin D supplements. In 2003, at the aged of 13, he was reviewed by the geneticist as his parents wanted to know about possible genetic risks if they were to have more children. The possibility of Blau syndrome was discussed and confirmed the following year with a mutation found in NOD2 gene. Both his parents were tested negative for the mutation. At the age of 14, he developed bilateral uveitis and papilloedema. MRI head did not show any features of raised intracranial pressure and hence the papilloedema was thought to be secondary to inflammatory infiltration. He required the addition of dexamethasone eye drops in addition to subcutaneous methotrexate 25mg weekly and prednisolone. Uncontrolled disease (eye, skin and joint manifestations) prompted us to commence anakinra 100mg subcutaneously daily. By three months his joints had improved as had his level of physical activity at school, but the skin and eyes had not responded. At six months, the uveitis had responded and he was able to wean off prednisolone, but his skin rash persisted. At 12-months he suffered a relapse and developed nausea with methotrexate, prompting a switch to ciclosporin at 2mg/kg. Higher doses caused renal impairment. The combination of anakinra and cyclospirin controlled his disease util he attended follow-up at the age of 21. He was found to have widespread asymptomatic synovitis. An ultrasound of his hands demonstrated moderate synovial thickening affecting all of the MCP’s with obvious erosive changes, but no evidence of neovascularisation. At this time, the ciclosporin had to be discontinued because of renal impairment. Following this discontinuation, he developed widespread arthritis involving his knees, elbows, metacarpophalangeal joints and shoulder. He also required a left cataract operation and an intra-vitreal bevacizumab for a relapse of his granulomatous eye disease. We commenced Infliximab 5mg/kg every eight weeks along with prednisolone. Nineteen months later, he is asymptomatic. His CRP has dropped from 47 mg/L to 3 mg/L (normal range 0-10 mg/L). He is currently working full time and off prednisolone.

**Discussion:** Blau syndrome is a rare autosomal dominant autoinflammatory disease characterised by early onset granulomatous arthritis, uveitis and rash. Dr Blau first described it in 1985 in family members over four generations. Then in 2001, Miceli-Richard et al discovered the mutations in NOD2 (nucleotide-binding oligomerization domain protein-2) that is also called CARD15 (caspase activation recruitment domain-containing protein-15). The prevalence is less than 1 in 1,000,000. The manifestations of Blau syndrome usually begin in early childhood with skin rashes being typically the earliest sign. The rashes are most commonly seen as brown-red flat-topped papules. It is slightly scaly, discrete and described as tapioca grain-like. Arthritis is often symmetrical and polyarticular involving mainly the wrists, ankles, knees and proximal interphalangeal (PIP) joints, commonly with marked tenosynovitis. Flexion contracture of the PIP joints leading to camptodactyly can develop during the disease. The arthritis can cause joint deformity leading to disability without appropriate treatment. Granulomatous uveitis is usually the last feature to develop and 50% can develop cataracts as in our patient. It can also lead to glaucoma and blindness. Other clinical manifestations include fever, malignant hypertension, granulomatous large vessel vasculitis and granulomatous inflammation of the liver, kidneys and lung. The treatment of Blau syndrome is challenging. It is usually treated initially with oral prednisolone alone or in combination with methotrexate, ciclosporin or mycophenolate mofetil. Interleukin 1β receptor antagonists such as anakinra and canakinumab have been used and the outcome has been variable. The defect in NOD2/CARD15 gene causes increased activity of NF-kB, which causes an upregulation of TH1 cytokines, amongst which TNF-alpha is the most targetable one. In 2016, an individual funding request for anti TNF-alpha was applied for our patient. There have been a few case reports of the use of anti TNF-alpha in the treatment of Blau syndrome with good response.

**Key Learning Points:** Blau syndrome is rare and diagnosis needs to be considered in patients who present in childhood with skin rashes as often the first presentation followed by arthritis and uveitis. A skin biopsy is helpful in diagnosis and hence should be considered as part of initial investigation. Genetic testing for NOD2 and counselling is required. The input from multiple specialties is important in the management of patients with Blau syndrome. As the disease is often poorly controlled with standard DMARDs, consideration should be given to treatment with TNF-alpha inhibitors or interleukin 1β receptor antagonists if steroids cannot be weaned on methotrexate alone.

**Disclosure: C. Yong:** None. **C. Mukhtyar:** None. **K. Armon:** None.

